# Actin-Interacting Protein 1 Contributes to Intranuclear Rod Assembly in *Dictyostelium discoideum*

**DOI:** 10.1038/srep40310

**Published:** 2017-01-11

**Authors:** Hellen C. Ishikawa-Ankerhold, Wioleta Daszkiewicz, Michael Schleicher, Annette Müller-Taubenberger

**Affiliations:** 1 Department of Cell Biology (Anatomy III), Biomedical Center, LMU Munich, 82152 Planegg-Martinsried, Germany

## Abstract

Intranuclear rods are aggregates consisting of actin and cofilin that are formed in the nucleus in consequence of chemical or mechanical stress conditions. The formation of rods is implicated in a variety of pathological conditions, such as certain myopathies and some neurological disorders. It is still not well understood what exactly triggers the formation of intranuclear rods, whether other proteins are involved, and what the underlying mechanisms of rod assembly or disassembly are. In this study, *Dictyostelium discoideum* was used to examine appearance, stages of assembly, composition, stability, and dismantling of rods. Our data show that intranuclear rods, in addition to actin and cofilin, are composed of a distinct set of other proteins comprising actin-interacting protein 1 (Aip1), coronin (CorA), filactin (Fia), and the 34 kDa actin-bundling protein B (AbpB). A finely tuned spatio-temporal pattern of protein recruitment was found during formation of rods. Aip1 is important for the final state of rod compaction indicating that Aip1 plays a major role in shaping the intranuclear rods. In the absence of both Aip1 and CorA, rods are not formed in the nucleus, suggesting that a sufficient supply of monomeric actin is a prerequisite for rod formation.

Nuclear rods consist of bundles of filamentous actin and were first identified in the nuclei of *Dictyostelium discoideum* amoebae and in HeLa cells after treatment with high concentrations of dimethyl sulfoxide (DMSO)^1^,^2^,^3^,^4^. Owing to different stress conditions, rods have been described in a variety of cell types, and are involved in a number of neurodegenerative diseases in humans^5^,^6^,^7^,^8^. In addition, intranuclear rods have been found in heat-shocked neurons, and in Huntingtin mutant or silenced cells suggesting a role in Huntington’s disease (HD)^9^. Furthermore, intranuclear actin rods were identified as hallmarks in muscle cells of patients with intranuclear rod myopathy (IRM), a specific form of nemaline myopathy^10^,^11^,^12^,^13^,^14^.

The exact protein composition of the rods and the mechanisms that trigger their formation is unclear. To date, especially cofilin as an actin-associated protein has been identified in DMSO-induced nuclear actin rods^15^,^16^. Cofilin is a protein located primarily in the cytoplasm, although it translocates into the nucleus together with actin in response to various stress conditions. The functional roles of cofilin-actin rods in the nucleus remain to be elucidated, but the general assumption is that the formation of nuclear rods constitutes an option to reduce energy consumption due to a shut-off of actin-treadmilling, and thus provides a protective mechanism for the cell.

In the present work, in addition to actin and cofilin further proteins were identified as constituents of intranuclear rods including actin-interacting protein 1 (Aip1), coronin (CorA), 34-kDa actin-bundling protein B (AbpB), and filactin (Fia). We have analysed the spatio-temporal recruitment of these proteins into intranuclear rods, and describe the dynamics of intranuclear rod assembly and disassembly. Our results indicate that Aip1 plays a crucial role for intranuclear rod configuration and compaction.

## Results

### Temporal progression of intranuclear rod assembly in *Dictyostelium* cells

**Figure 1:**
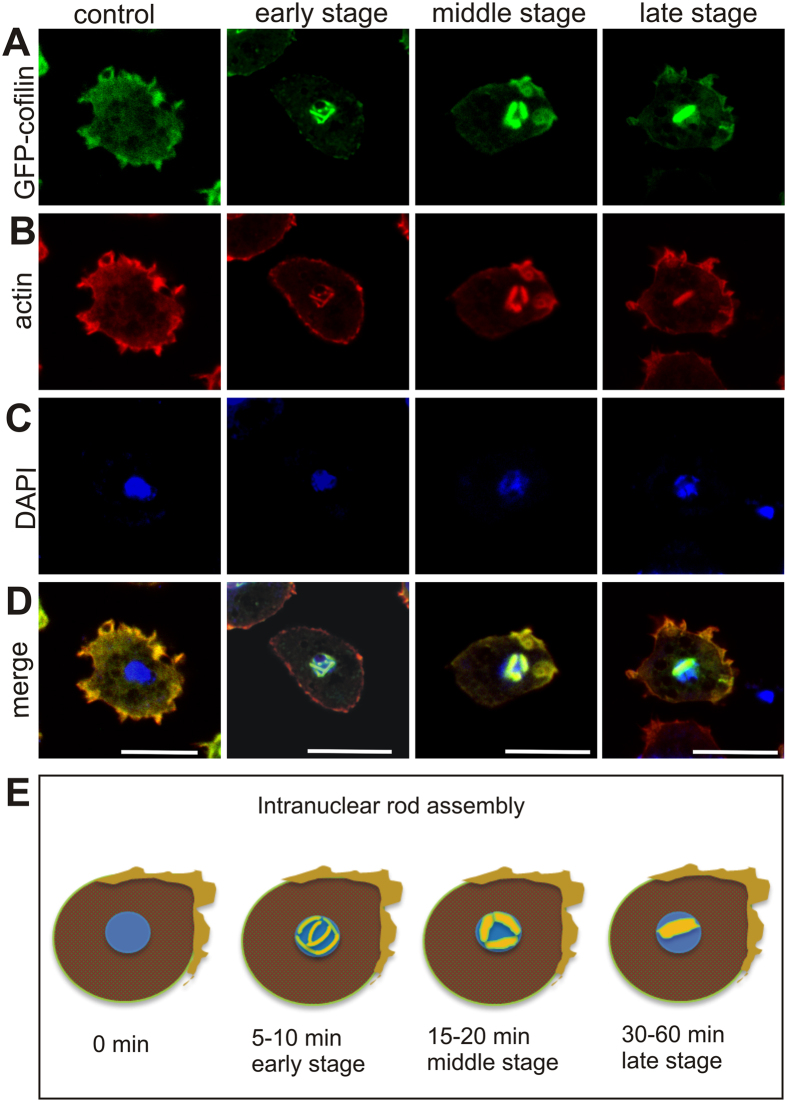
Stages of intranuclear rod assembly. (**A**) *Dictyostelium* cells expressing GFP-cofilin (green) were induced to form nuclear actin rods by treatment with 5% DMSO, and fixed after 5, 10, 15, 20, 30 and 60 minutes. (**B**) Cells were labeled with monoclonal mouse anti-actin and secondary goat anti-mouse Alexa Fluor-563 labeled antibodies to visualize actin (red). (**C**) Nuclear DNA was stained with DAPI (blue). (**D**) Merged images. The assembly of rods was classified according to the stage of actin-cofilin filaments arrangement. (**E**) Scheme depicting stages of intranuclear rod assembly. In the early stage (5–10 min) of DMSO treatment, the rods are thin and dispersed throughout the nucleoplasm. In the middle stage (15–20 min), rods start to compact close to the nuclear membrane. In the late stage (30–60 min), rods form thick, bar-like structures. Scale bars are 10 μm.

Live-cell imaging recordings of *Dictyostelium* cells expressing GFP-cofilin treated with 5% DMSO enabled us to follow the progression of rod assembly in the nucleus over time (Fig. 1 and Movie 1). Formation of rods started 5 min after onset of the DMSO treatment (Fig. 1, early stage), and showed maximal compaction after 30 to 60 min of treatment (Fig. 1, late stage). Initially, short, needle-like actin-cofilin aggregates were detectable in the cytoplasm, and after 5 min of DMSO treatment these short assemblies disappeared from the cytoplasm, and rods started to aggregate inside the nucleus adjacent to the inner nuclear membrane (early stage after 5 to 10 min). Subsequently these needle-like structures compacted into bundles (middle stage after 15–20 min; see Movie 2), and finally formed a dense, bar-shaped configuration within the nucleus (late stage 30 to 60 min; see Movie 3).

### Spatiotemporal recruitment of rod proteins

**Figure 2:**
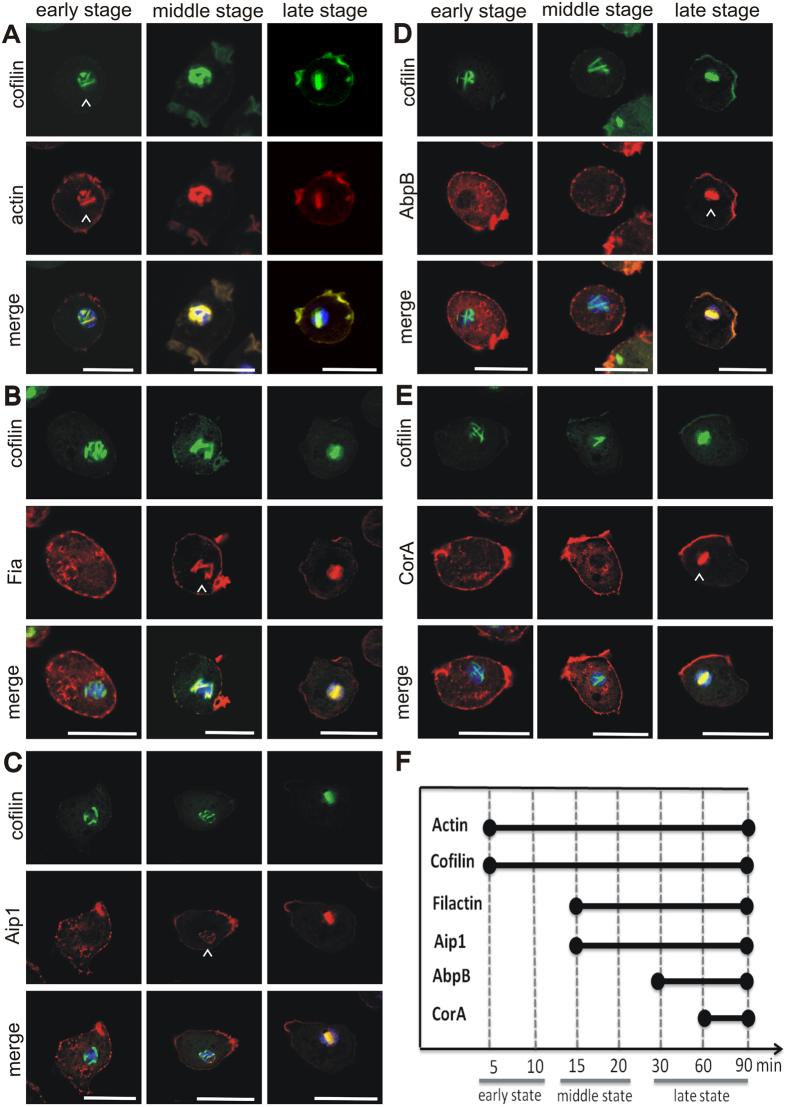
Intranuclear rod composition. *Dictyostelium* wild-type cells were treated with 5% DMSO for different time periods (5, 10, 15, 20, 30, 60 and 90 min) to induce the formation of nuclear rods. Then, cells were fixed and labeled with rabbit polyclonal antibodies against cofilin and Alexa Fluor-488 labeled secondary antibodies (green), and monoclonal mouse antibodies directed against (**A**) actin, (**B**) filactin (Fia), (**C**) Aip1, (**D**) 34-kDa actin-bundling protein (AbpB), or (**E**) coronin A (CorA), and Alexa Fluor-594-labeled secondary antibodies (red). Nuclei were visualized by staining with DAPI (blue). Arrowheads indicate the first appearance of nuclear rods. Bars are 10 μm. (**F**) Time course of protein recruitment to nuclear rods. *Dictyostelium* cells expressing GFP-cofilin were treated with 5% DMSO for different time periods, and were then fixed and labeled with antibodies directed against actin, Fia, Aip1, AbpB, and CorA as shown in (**A–E)**. The samples for each time point and antibody were analysed for the presence of the individual proteins in the nuclear rods. The experiment was repeated three times and more than 80 cells per sample were inspected.

Next, we attempted to test whether, in addition to actin and cofilin, other cytoskeletal proteins are associated with nuclear rods. For this, we employed immunofluorescent labelling and tested specific antibodies directed against a number of cytoskeletal proteins after induction of nuclear rods (Table 1, Fig. 2). This analysis confirmed not only actin and cofilin as constituents of intranuclear rods, but also revealed the presence of four other actin-binding proteins including coronin (CorA), actin-interacting protein 1 (Aip1), the actin variant filactin (Fia), and the actin-bundling protein B (AbpB). Other cytoskeleton-associated proteins like α-actinin or capping proteins were not detected by this approach (Table 1, Suppl. Fig. 1).

In an alternative approach to analyse the protein composition of nuclear rods, we purified the rods (Suppl. Fig. 2), and subjected them to mass spectrometry (Suppl. Fig. 3). The results showed that rods are not only composed of actin and cofilin, but also confirmed the presence of CorA, Aip1, AbpB and Fia whereas other actin-binding or cytoskeleton-associated proteins were not detectable in intranuclear actin rods (Table 1).

The identification of actin-binding proteins that previously have not been described in nuclear rods prompted us to characterize the spatiotemporal recruitment of these proteins during rod assembly (Fig. 2). At the early stage of rod assemble (first 5–10 min after rod induction), only actin and cofilin were detectable (Fig. 2A). At the middle stage (15–20 min), Fia (Fig. 2B) and Aip1 (Fig. 2C) were recruited. Only at the late stage (after 30 min), when nuclear rods matured into a thick, bar-shaped conformation, AbpB (Fig. 2D) and CorA (Fig. 2E) became associated. The time course of protein recruitment into rods is summarized in Fig. 2F.

### Aip1 plays an essential role in rod assembly

**Figure 3:**
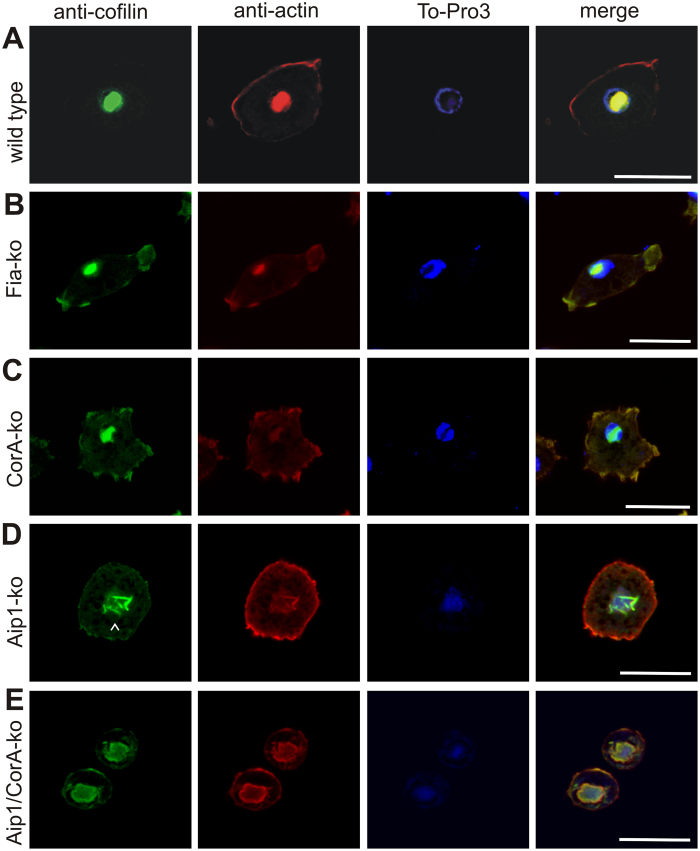
Aip1 is essential for the spatio-temporal control of intranuclear rod assembly. *Dictyostelium* wild-type AX2, CorA-null, Aip1-null or Aip1/CorA-null cells were induced to form actin rods in the nucleus by treatment with 5% DMSO for different time periods. Cells were fixed and immuno-labeled with antibodies directed against cofilin (green) and actin (red), and nuclei were stained with TO-PRO-3 (blue). After 60 min of DMSO treatment, the intranuclear rods compacted into bundle-shaped assemblies in (**A**) wild-type cells, (**B**) Fia-null cells, and (**C**) CorA-null cells. (**D**) In the absence of Aip1, the formation of nuclear rods is imperfect. Even after prolonged treatment with DMSO (90–120 min), only a needle-like configuration is achieved, characteristic of the middle stage in wild-type cells as indicated by the arrowhead. (**E**) Cells lacking both Aip1 and CorA are unable to form compact intranuclear actin rods, but assemble actin and cofilin close to the nuclear membrane. Scale bars are 10 μm.

**Figure 4:**
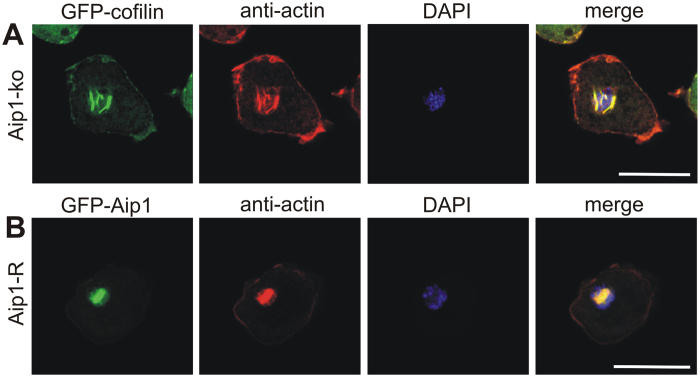
Impaired intranuclear rod formation can be rescued by expression of GFP-Aip1. (**A**) Aip1-null cells expressing GFP-cofilin, and (**B**) Aip1-null cells expressing a functional rescuing construct^17^, GFP-Aip1, were induced to form actin rods in the nucleus by treatment with 5% DMSO for 60 min. Cells were fixed and immuno-labeled with antibodies directed against actin (red), and nuclei were stained with DAPI (blue). Scale bars are 10 μm.

The availability of knockout mutants lacking individual rod constituents enabled us to analyse the sequential pattern of intranuclear rod assembly in more detail (Fig. 3). In the absence of Fia (Fig. 3B), or CorA (Fig. 3C), bar-shaped rods were still detectable suggesting that these two proteins are not essential for intranuclear rod compaction and maturation. However, in mutants lacking Aip1, the typical nuclear compacted bar shape characteristic for rods of the late stage was never observed even after prolonged treatment with DMSO for up to 2 h (Fig. 3D). In the absence of Aip1, rod assembly was halted at the middle stage but never completed to compacted rods indicating an essential role for Aip1 in the maturation process of nuclear rods. The deficiency of rod compaction in Aip1-mutants could be rescued by expression of GFP-Aip1 (Fig. 4), a construct that was shown previously to rescue functional defects in Aip1-null mutants^17^. Interestingly, in the absence of both Aip1 and CorA, needle-like or bar-shaped rods were not detectable in the nucleus (Fig. 3E). This suggests that CorA acts synergistically to complement Aip1 functions in nuclear rod assembly, and shows that the formation of rods, in addition to actin and cofilin, is dependent on proteins that provide a sufficient pool of monomeric actin.

### Rod assembly by nucleation of monomeric actin

**Figure 5:**
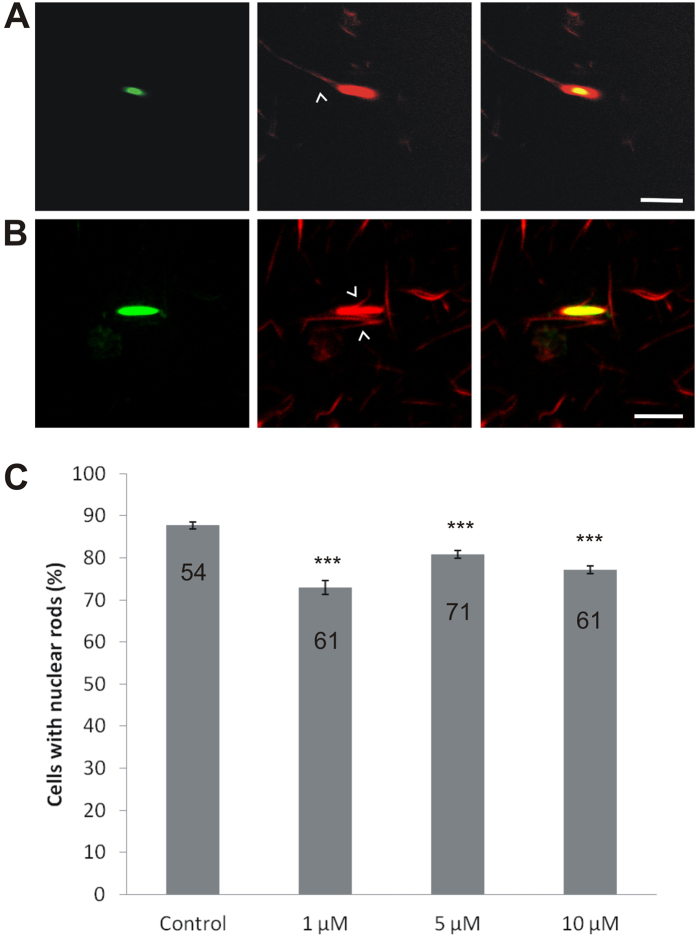
Monomeric actin can polymerize along the rod extremities and core sides. Cells expressing GFP-cofilin were induced to form actin rods in the nucleus by treatment with 5% DMSO for 1 h. After induction, the cells were lysed, and rods were extracted and purified as described in material in methods. (**A**,**B**) Isolated rods were mixed with monomeric G-actin and subjected to an actin polymerization assay. After 1 h, TRITC-phalloidin was added to label filamentous actin. Actin polymerizes at the isolated rod extremities as indicated by the arrow (**A**), and also at the sides of rods as shown in (**B**). Scale bars are 5 μm. (**C**) Treatment with cytochalasin D does not substantially inhibit nuclear rod formation. Treatment with 1, 5 or 10 μM of cytochalasin D significantly reduced rod assembly by around 10 to 17% compared to untreated control cells. The numbers indicate the number of counting areas (50 cells each). Data are presented as mean ± S.E.M. Statistical significance by unpaired t-test two-tailed is shown (***p < 0.0001), p values less than 0.05 were considered significant. GraphPad PRISM software was used for statistical analysis.

To analyse whether rods can serve as nuclei for new filament growth, rods were purified as described in Methods (see also Suppl. Fig. 2). The isolated rods were incubated with G-actin and subjected to an actin polymerization assay. Newly polymerized actin was visualized by addition of TRITC-phalloidin and fluorescence microscopy. Actin filament growth was observed both at the rods extremities and along the sides, indicating that rods contain uncapped filament ends throughout the whole structure that serve as nucleation sites for actin assembly (Fig. 5A,B; Movies 4 and 5).

To get more insights into the mechanisms of rod formation, we treated GFP-cofilin expressing cells with different concentrations of cytochalasin D (CytD), a drug that inhibits barbed-end growth. Inhibition of actin assembly by CytD considerably reduced nuclear rod formation by 17% (1 μM), 10% (5 μM), and 13% (10 μM) compared to untreated controls (Fig. 5C). This observation suggests that rods form not only by elongation of barbed ends, but also by nucleation along other binding sites, as shown in Fig. 5B and Movie 5, probably induced by the action of cofilin as previously reported^18^,^19^.

In our studies employing fixed cells, nuclear rods were labelled with anti-actin antibodies to verify the presence of actin in nuclear rods (Fig. 2A). However, an open question is whether actin in intranuclear rods adopts a special filamentous state. Phalloidin is known to bind to actin filaments due to its conformational interaction with at least three F-actin subunits in the groove of the two-stranded helix^20^. We have shown that TRITC-phalloidin also stains GFP-cofilin-labelled intranuclear rods. To investigate whether phalloidin incorporates also into cytoplasmic actin rods, rods were induced by the addition of sodium azide. In contrast to nuclear rods, cytoplasmic rods were not stainable with phalloidin indicating a different conformation of actin (Suppl. Fig. 4).

### Rod dynamics investigated by fluorescence recovery after photobleaching (FRAP)

**Figure 6:**
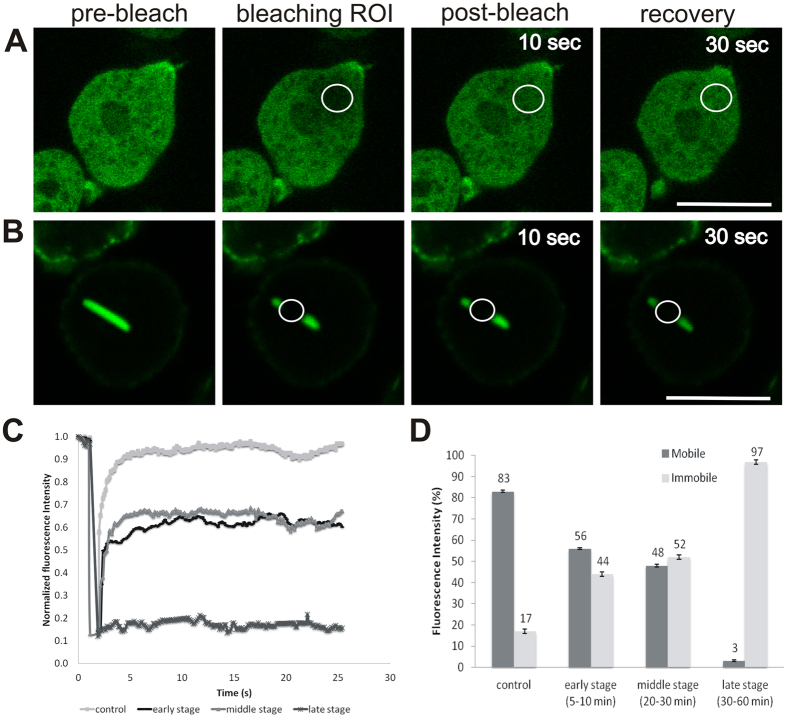
Intranuclear rod dynamicity analysed by fluorescence recovery after photobleaching (FRAP). (**A**) In untreated GFP-cofilin cells (control), the recovery after a bleaching event is quick and the mobile fraction (Mf) is high (83%) indicating a highly dynamic mobile fraction. (**B**–**D**) When the intranuclear rods start to form after 5–10 min of DMSO treatment (early stage), the recovery after a bleaching event is slower with a mobile fraction of 56%. The mobile fraction decreases during further rod formation. In the middle stage, the mobile fraction comprises 48% indicating an increasingly restricted protein dynamics. At the late stage, when the bundles compacted into bar-shaped rods almost no GFP-cofilin is available in the nucleoplasm (Mf = 3%). (**B**) Shows one example of bleaching at the late stage. (**C**) Depicts bleaching curves corresponding to the defined stages. (**D**) The histogram summarizes the relative proportion of mobile and immobile fractions during the different stages of rod assembly. Error bars represent standard errors.

The dynamicity of nuclear rod proteins was analysed by FRAP experiments, which demonstrated that the internal fluctuation of cofilin in nuclear rods decreased during rod assembly (Fig. 6A–C). At the early stage of rod formation (5–10 min after induction), the mobility of cofilin is around 56%, and the rods were dispersed in the nucleoplasm. The mobility decreased to 48% in the middle stage after 15–20 min of induction when the rods started to compact. Only about 3% of GFP-cofilin was found to be mobile in the late stage after 30–60 min of induction when thick bars were already formed (Fig. 6D). This indicates extremely little exchange of proteins in mature nuclear rods.

### Intranuclear rod disassembly

After 1 h of 5% DMSO treatment, about 83% of the cells were in the late stage showing compacted thick bar-shaped rods inside the nucleus. When the stress stimulus was removed, nuclear rods disassembled quickly, and the cells recovered to a normal state within 30 min. To quantify the disassembly times, wild-type and knockout cells (Aip1-ko, CorA-ko, and Fia-ko) were treated with 5% DMSO for 1 h, washed twice to remove the chemical stimulus and incubated with medium for recovery, and then were fixed for immunofluorescence after different time periods. Images were taken to quantify the rod disassembly after 5, 10, 20 and 30 min. The time course of rod disassembly showed that in wild-type cells almost all actin rods were disassembled 30 min after removal of the DMSO (Suppl. Fig. 5). In cells lacking either CorA or Fia, rod disassembly was significantly delayed indicating a role for these actin interactors in dismantling of rods. In Aip1-null cells, rod disassembly was faster than in wild type, however this result is not unexpected as rods are not fully matured in the mutant.

## Discussion

Intranuclear rods are of medical relevance because they have been associated with variants of nemaline myopathies^10^,^21^, HD^22^, and certain other neurodegenerative diseases in humans^5^. Here, we have analysed the formation and composition of intranuclear rods in *Dictyostelium discoideum*, a molecular model organism that is increasingly being used to explore the cellular basics of neurological disorders^23^. In fact, the first descriptions of intranuclear rods were from work using different *Dictyostelium* species. Electron microscopic studies demonstrated the formation of huge microfilament bundles in the nuclei of interphase cells treated with DMSO^3^. These bundles were described as structures of approximately 3 μm in length and 0.85 μm in width. Intranuclear rods then were also found after DMSO treatment in other cell types like HeLa cells^1^, and subsequently it was shown that these structures not only contain actin but also cofilin^15^.

Actin rods have been described previously to occur in *Dictyostelium* during the formation of spores^24^. Sporulation is a stage of dormancy to survive harsh and unpleasant environmental conditions. In spores the cellular metabolism is stalled, but can be rapidly reactivated even after very long resting periods. However, the actin rods described in spores were formed both in the cytoplasm and in nucleus. The ultrastructural analysis of these rods showed a hexagonal arrangement of actin tubules. The disassembly of these rods followed a slower time scale compared to the disassembly of nuclear actin rods described in this study (Suppl. Fig. S5), indicating that different modes of disassembly may exist.

In the present study, we have analysed the formation of intranuclear rods in more detail using methods that are not applicable in studies of the diseased state in affected humans. We have classified three stages of rod assembly. (1) At the early stage, the actin/cofilin filaments form thin, needle-like structures that appear randomly distributed adjacent to the inner nuclear membrane. (2) In the middle stage, after 15 minutes of DMSO treatment, the intranuclear bundles gradually compact. (3) In the late stage, after 30–60 minutes of treatment, the bundles coalesce and form a central, thick bar with the extremities extending close to the nuclear membrane, but not traversing the nuclear membrane (Movie 1). This late phase corresponds to the stages that have been analysed previously by electron microscopy^3^.

The studies on the composition and assembly mechanism of nuclear rods aim to understand the causes of the diseased state. Mass spectrometry of purified actin rods and immunofluorescence labelling using specific antibodies identified the presence of a specific set of actin-binding proteins that have previously not been described to be involved in intranuclear rod assembly (Fig. 2). Our analysis of the spatiotemporal appearance showed that filactin (Fia), actin-interacting protein 1 (Aip1), coronin (CorA) and the actin-bundling protein (AbpB) are assembled in a sequential manner during the formation of intranuclear rods. Actin and cofilin are the only proteins present at the early stage and were identified at all stages of rod assembly (Figs 1 and 2), Fia and Aip1 become associated at the middle stage, and CorA and AbpB are added at the late stage (Fig. 2).

Filactin is a novel actin variant identified only in *Dictyostelium*^25^. Its function is not well understood, but a role in actin depolymerization was suggested^26^. Both, Aip1 and CorA, are involved in the regulation of actin filament disassembly and turnover. CorA functions in enhancing cofilin activity by promoting recycling of actin monomers to support the continuous actin assembly at the cell front^27^,^28^. Aip1 enhances the activity of ADF (actin depolymerizing factor)/cofilin in filament fragmentation by its barbed-end capping activity that prevents elongation and re-annealing of the severed filaments^29^, and was shown to maintain the intracellular pool of monomeric actin^30^. In *Dictyostelium* cells, the localization of Aip1 is very similar to cofilin, and it colocalizes with actin filaments in dynamic structures such as leading edges of motile cells, phagocytic cups, and macropinosomes^31^. When cells are deficient in both, Aip1 and CorA, the content of filamentous actin is highly increased causing a number of defects linked to disturbed actin dynamics^32^. AbpB is a calcium-regulated actin-crosslinking protein of *Dictyostelium*^33^,^34^. AbpB is recruited only during the late stage of intranuclear rod formation and may contribute to bundling and compaction of the rods. Due to its bundling activity, AbpB may be important for cross-linking actin filaments during the late stage. Previous studies have shown that AbpB also associates with paracrystalline structures of actin filaments, and that AbpB is involved in Hirano bodies’ formation that were reported as hallmarks of a variety of neurodegenerative diseases^35^,^36^,^37^. Other proteins like those known to mediate actin crosslinking such as α-actinin were not detected and are most probably not involved in the formation of intranuclear rods in *Dictyostelium* (Suppl. Fig. 1, and Table 1). This result is in contrast to findings in C2C12 myoblasts carrying specific mutations in the skeletal actin gene *ATCA* that revealed the presence of α-actinin in intranuclear aggregates^14^, and implicates that different types of nuclear actin rods may exist.

The identification of proteins that were previously not reported to constitute nuclear actin rods, prompted us to analyse null mutants of these proteins for their contribution to intranuclear rod formation. Mutants lacking CorA or Fia formed intranuclear rods indistinguishable from wild-type cells, but in the absence of Aip1, the assembly of intranuclear rods was strongly affected (Fig. 3). Rods appeared only as needle-like structures characteristic for the middle stage of rod maturation. This suggests that the primary function of Aip1 in rod formation is to provide sufficient amounts of actin monomers that shuttle into nucleus. In addition, Aip1 may also act to cap actin filaments and to stabilize formation of rod by clipping the filaments ends together in order to form the compact bar-shaped rods of the mature stage. Mutants lacking both Aip1 and CorA were even more severely disturbed in intranuclear rod formation suggesting that efficient generation and delivery of actin monomers from cytoplasmic pools into the nucleus is an essential prerequisite for rod formation by *de novo* polymerization of actin in the nucleus. The notion that Aip1 may enhance or modulate cofilin-mediated activities on actin dynamics during nuclear rod formation has been proposed only recently^38^.

For the analysis of rod growth and dynamics, we have used either specific antibodies or cell lines expressing GFP-fusion proteins. However, in live-cell imaging studies employing the actin marker Lifeact-GFP^39^, nuclear rods were not visualized implying that Lifeact-GFP is either not re-located into the nucleus or is excluded from rod formation. Intranuclear actin rods, but not cytoplasmic rods (Suppl. Fig. 4), are stainable with phalloidin, which indicates that these assemblies are structurally similar to cortical actin (Fig. 5A). This suggests that nuclear and cytoplasmic rods differ in their structural arrangement and/or composition, an issue that requires further attention in future studies. In an actin assembly assay using purified GFP-cofilin rods, we showed that rods can grow by actin polymerization at the filament extremities, but also by actin polymerization at the core sides (Fig. 5B, Movie 5). Interestingly, the application of low doses of CytD, a barbed-end capping drug to inhibit actin polymerization, caused a reduction of nuclear rods formed inside nuclei, but was not sufficient to block rod formation as a whole (Fig. 5B,C). This implies that rods grow not only at the barbed ends, but potentially also by nucleation along filament sides as indicated by our image analyses (Movie 5).

Rod formation may provide a protective mechanism of the cells under stress conditions. To get more insights into the dynamics of rods, FRAP experiments were performed using GFP-cofilin expressing cells. In the mature stage, GFP-cofilin is almost immobile (Fig. 6B–D) indicating that rods are very stable structures with negligible exchange of cofilin in the compacted stage. When the stress stimulus is removed, nuclear rods disassemble relatively fast. Within 30 minutes after DMSO removal, about 90% of the rods have disappeared (Suppl. Fig. 5). Our results show that in the absence of CorA or Fia the disassembly of intranuclear rods is impaired, suggesting that both proteins play a role in the dismantling of matured rods.

In conclusion, the present study shows that not only actin and cofilin are implicated in intranuclear rod formation in *Dictyostelium*, but also Aip1, CorA, FiaA and AbpB. Most notably, our data indicate that Aip1 is important to provide the actin monomer supply required for intranuclear rod assembly. Aip1 and CorA were reported previously to synergistically control the turnover of filamentous actin. The finding that mutants lacking Aip1, or Aip1 as well as CorA, were severely disturbed in intranuclear rod formation supports the notion that efficient generation and delivery of actin monomers from cytoplasmic pools into the nucleus is essential for nuclear rod formation. It will be interesting to examine whether Aip1, and possibly coronins, play a similar role in nuclear rod formation of human cells. A previous study analysing nuclear cofilin-actin rods in HD has already discussed defective actin turnover under stress conditions as one of the main causative disorders during the development of the disease^22^, and huntingtin and transglutaminase 2 were shown to be implicated in the pathogenic mechanism of HD. There is no question that different causes have to be considered which finally manifest in actin-cofilin rod assemblies and thus indicate a pathological state. Actin-binding proteins involved in the regulation of actin turnover add another facet to an insufficiently understood cellular state and may open new avenues to develop potential therapeutic strategies to treat nuclear rod diseases.

## Methods

### Strains, culture conditions, and treatments

The axenic strain AX2-214 of *Dictyostelium discoideum* was used as wild type. Mutant strains lacking Aip1^17^, CorA^40^, CorA/Aip1^32^, filactin^26^, and a GFP-Aip1 expressing, rescued Aip1-null strain^17^ were described previously. Cells were grown in HL5 axenic medium (Formedium), or on SM plates together with bacteria at 21 °C.

For expression of GFP-cofilin under control of an actin-15 promoter, the full-length coding sequence of cofilin A (DDB_G0277833) was cloned into the *Cla*I site of the pDEX79 vector containing a G418 resistance cassette^41^. Cells expressing GFP-cofilin and/or mRFP-actin, were cultivated in the presence of antibiotics (10 μg/ml G418 and/or 10 μg/ml blasticidin). Expression levels of GFP-cofilin approximately equalled endogenous cofilin levels, and were not altered by treatment of the cells with DMSO (Suppl. Fig. 6).

For induction of nuclear rods, DMSO was diluted in HL5 medium to a final concentration of 5%. To induce cytoplasmic rods, sodium azide was added to HL5 medium to a final concentration of 10 mM. Cytochalasin D (Sigma) was diluted in HL5 medium to final concentrations of 1, 5, or 10 μM.

### Immunocytochemistry and antibodies

*Dictyostelium* cells were plated on round 12-mm glass coverslips, and after 30 min cells were fixed in methanol at −20 °C for 15 min, or with 15% picric acid/2% paraformaldehyde in 10 mM PIPES, pH 6.0, for 20 min, and post-fixed with 70% ethanol for 10 min. Then, the cells were washed three times in PBS, once with 10 mM PIPES, and twice with PBS/1% glycine, and incubated in blocking buffer (PBS plus 2% bovine serum albumin) for 1 h at room temperature (RT). After blocking, the cells were washed three times with PBS and incubated with primary antibodies for 1 h at RT or overnight at 4 °C, followed by the incubation with secondary antibodies and TO-PRO3 iodide or DAPI, to stain DNA, for 1 h at RT. After immunostaining, samples were washed three times in PBS and embedded using Gelvatol or Dako (Agilent Technologies) mounting media. For assaying rod disassembly, cells were washed twice with medium to remove the DMSO stimulus, and fixed after the indicated periods of time.

For the generation of antibodies specific for *Dictyostelium* cofilin, the protein was expressed in bacteria. The cDNA encoding *cofA* was cloned via *Bam*HI and *Not*I into a pGEX-6P1 vector (GE Healthcare Life Sciences), expressed in *E. coli* (Rosetta, Novagen), and glutathione S transferase (GST)-tagged cofilin was purified via glutathione sepharose (GE Healthcare Life Sciences). Polyclonal antibodies against *Dictyostelium* cofilin (pAb 496) were obtained by immunizing a female New Zealand White rabbit (Charles River Laboratories) with the recombinant protein together with the adjuvant Gerbu 100 (Gerbu Biochemicals).

Other antibodies used in the study were specific for: actin (mAb Act1)^42^, Aip1 (mAb 246-153-2 and 246-404-2)^17^, coronin (mAb 176-3-6)^32^, filactin (mAb 3S-55-4)^26^, LimE (mAb 310-111-3) (kind gift of Günther Gerisch), profilin I (mAb 153-246-10)^43^, profilin II (mAb 174-380-3), α-actinin (mAb 47-18-9)^44^, β-tubulin (mAb WA3) (kind gift of Ursula Euteneuer), 14-3-3 protein (pAb 392)^45^, myosin II (mAb 56-396-5)^46^, Cap32 (mAb 43-442-1)^47^, Cap34 (mAb 108-19-94), severin (mAb 102-200-1)^48^, and AbpB (34 kDa actin-bundling protein) (mAb 159-291-1)^49^.

Secondary antibodies used in the study were Cy3-conjugated goat anti-mouse, Alexa Fluor-488, -563, or -94 goat anti-mouse or anti-rabbit IgG (Molecular Probes). For some experiments, TRITC-phalloidin and/or TO-PRO3 iodide (Molecular Probes) or DAPI were added together with the secondary antibody.

### Imaging and FRAP (Fluorescence recovery after photobleaching) assay

Images were recorded using either a LSM 510 Meta confocal laser scanning microscope (Carl Zeiss Microscopy), or a LSM 880 AiryScan laser scanning microscope (Carl Zeiss Microscopy) equipped with PlanApo 63x/1.4 oil immersion objectives. GFP or Alexa Fluor-488, Cy3, Alexa Fluor-546 or -594, or TRITC, TO-PRO3, or DAPI were excited at 488, 556, 633 or 405 nm, respectively. Live-cell imaging experiments were performed as described^41^. Cells were recorded for about 5 min prior addition of DMSO.

For FRAP, GFP-cofilin-marked intranuclear rods in living cells were photobleached using the 488 nm-excitation laser line of the LSM 510 confocal microscope (100% intensity, 20 interactions) as described previously^50^. For quantification, each image was normalized by using the total fluorescence intensity in a region outside the FRAP (bleached) area to correct for any photobleaching caused by the image acquisition itself. The recovery rate of the fluorescence signal over time was monitored and used to determine the values of mobile and immobile fractions. The mobile fraction was determined by the recovering portion of the fluorescence, and the immobile fraction was the residual gap that was not recovering as compared to the pre-bleach situation.

### Purification of nuclear actin rods

GFP-cofilin expressing cells grown to confluence were stressed by addition of 5% DMSO for 1 h at 28 °C to induce the formation of intranuclear rods. After rod induction, 2 × 10^8^ cells were harvested by centrifugation at 200 × *g* and 4 °C for 5 min. The cell pellet was resuspended in 5 ml of NP-40 lysis buffer (50 mM HEPES, 50 mM Mg(OAc)_2_, 10% (w/v) sucrose, and 2% (v/v) Nonidet P40 (NP-40), pH 7.5. The suspension was passed twice through nucleopore filters (5 μm pore size) to enrich nuclei. The filtrate was checked for GFP-labelled nuclear rods using fluorescence microscopy, and centrifuged at 11,000 × *g* for 3 min. The pellet contained cellular debris and some aggregated rods, the supernatant contained the enriched rods (Suppl. Fig. 2A,B). Subsequently, rods were further purified by a two-step Optiprep (Sigma-Aldrich) gradient fractionation of 10 and 40% (w/v) using a TL-100 ultracentrifuge (Beckman Coulter) at 30,000 × *g* for 30 min at 4 °C. Rods were collected from the interface between the 10 and 40% Optiprep layers, and checked by microscopy (Suppl. Fig. 2C,D). Isolated rods were stable in different buffers: 50 mM HEPES pH 7.4, 100 mM phosphate buffer, pH 6.5, and 20 mM PIPES, pH 7.2.

Isolated rods were analysed by SDS-PAGE and Coomassie brilliant blue-R staining. Proteins bands were cut out from the gel and subjected to mass spectrometry (www.proteinanalytik.abi.med.uni-muenchen.de/service/index.html) (Suppl. Fig. 3A). Tryptic peptides were separated in an Ultimate 3000 high-performance liquid chromatography (HPLC) system (LC Packings). The effluent from the HPLC was directly electrosprayed into a linear trap quadrupole-Orbitrap mass spectrometer (Thermo Fisher Scientific). Proteins were identified using Mascot, and the databases Swissprot and Dictybase. Western blot analysis using antibodies directed against Aip1, CorA, actin, cofilin, and filactin confirmed the presence of proteins identified by mass spectrometry (Suppl. Fig. 3B).

### Actin polymerization assay

To test whether preformed actin rods act as nucleators, an actin polymerization assay was performed. 100 μl of isolated GFP-labeled rod suspension were incubated with purified and unlabeled G-actin (5 μM). Actin polymerization was induced by the addition of polymerization buffer (10 mM imidazole pH 7.2, 3 mM MgCl_2_, 1 mM Na-ATP, 0.2 mM CaCl_2_). After 1 h of incubation, a solution of TRITC-phalloidin was added to a final concentration of 50 μM for 1 h. Then, 50 μl of the stained solution were transferred to an observation dish coated with 0.1% collodion and subjected to confocal imaging.

## Additional Information

**How to cite this article**: Ishikawa-Ankerhold, H. C. *et al*. Actin-Interacting Protein 1 Contributes to Intranuclear Rod Assembly in *Dictyostelium discoideum. Sci. Rep.* **7**, 40310; doi: 10.1038/srep40310 (2017).

**Publisher's note:** Springer Nature remains neutral with regard to jurisdictional claims in published maps and institutional affiliations.

## Supplementary Material

Supplementary Dataset 1

Supplementary Movie 1

Supplementary Movie 2

Supplementary Movie 3

Supplementary Movie 4

Supplementary Movie 5

## Figures and Tables

**Table 1:** Proteins present and absent from nuclear rods.

Protein	MW kDa	Immunolabeling	Mass spectrometry
Filactin (Fia)	105	+	+
Actin-interacting protein 1 (Aip1)	67	+	+
Coronin (CorA)	50	+	+
Actin	42	+	+
Cofilin (CofA)	15	+	+
Actin binding protein 34 (AbpB)	33	+	+
LimE	20	—	—
Severin	39	—	—
Profilin I/II	13/12.7	—	—
α-actinin	97	—	—
ß-tubulin	51	—	‒
Capping protein (Cap32/34)	32/34	—	—
Myosin II	243	—	—
14-3-3	28	—	—
Coactosin	16	—	—
