# Supplementary material for: Actin-Interacting Protein 1 Contributes to Intranuclear Rod Assembly in Dictyostelium discoideum
Source: Sci Rep. 2017 Jan 11;7:40310. doi: 10.1038/srep40310 (PMC5225641; doi:10.1038/srep40310)
Supplement: Supplementary Dataset 1 [file srep40310-s1.doc]

**Supplementary Information**

**Actin-Interacting Protein 1 Contributes to Intranuclear Rod Assembly in *Dictyostelium discoideum***

**Hellen C. Ishikawa-Ankerhold^1^, Wioleta Daszkiewicz, Michael Schleicher, and Annette Müller-Taubenberger^2^**

**Supplementary Figures**

**
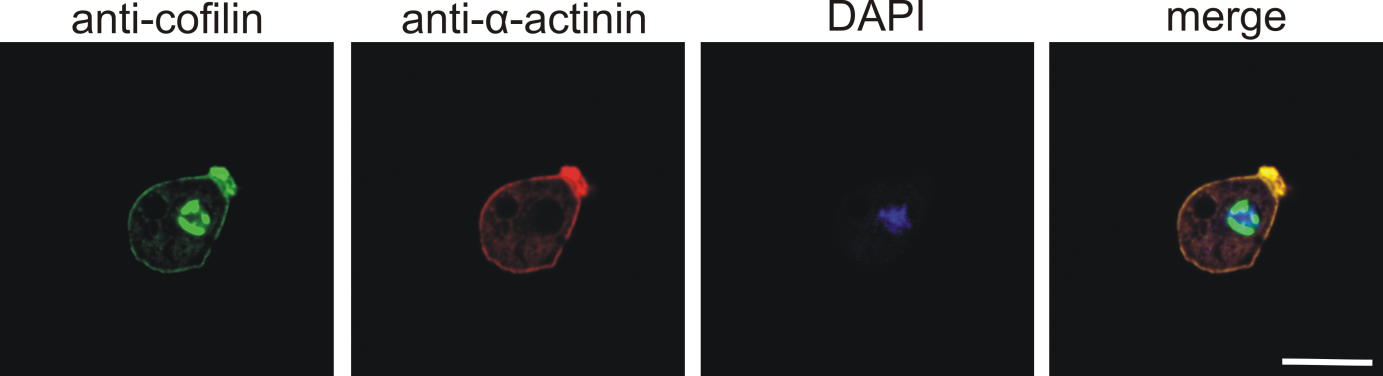
**

**Supplementary Figure 1.**  **α-actinin is not detectable in intranuclear rods.** *Dictyostelium* wild-type cells were induced to form intranuclear rods by treatment with 5% DMSO for 1 h. Then, the cells were fixed and labelled with rabbit polyclonal antibodies against cofilin and Alexa Fluor-488-labelled goat anti-rabbit secondary antibodies (green), and monoclonal mouse antibodies against α-actinin and goat anti-mouse Alexa Fluor-594-labelled secondary antibodies (red). Nuclear DNA was stained with DAPI (blue). Scale bars are 10 μm.

**
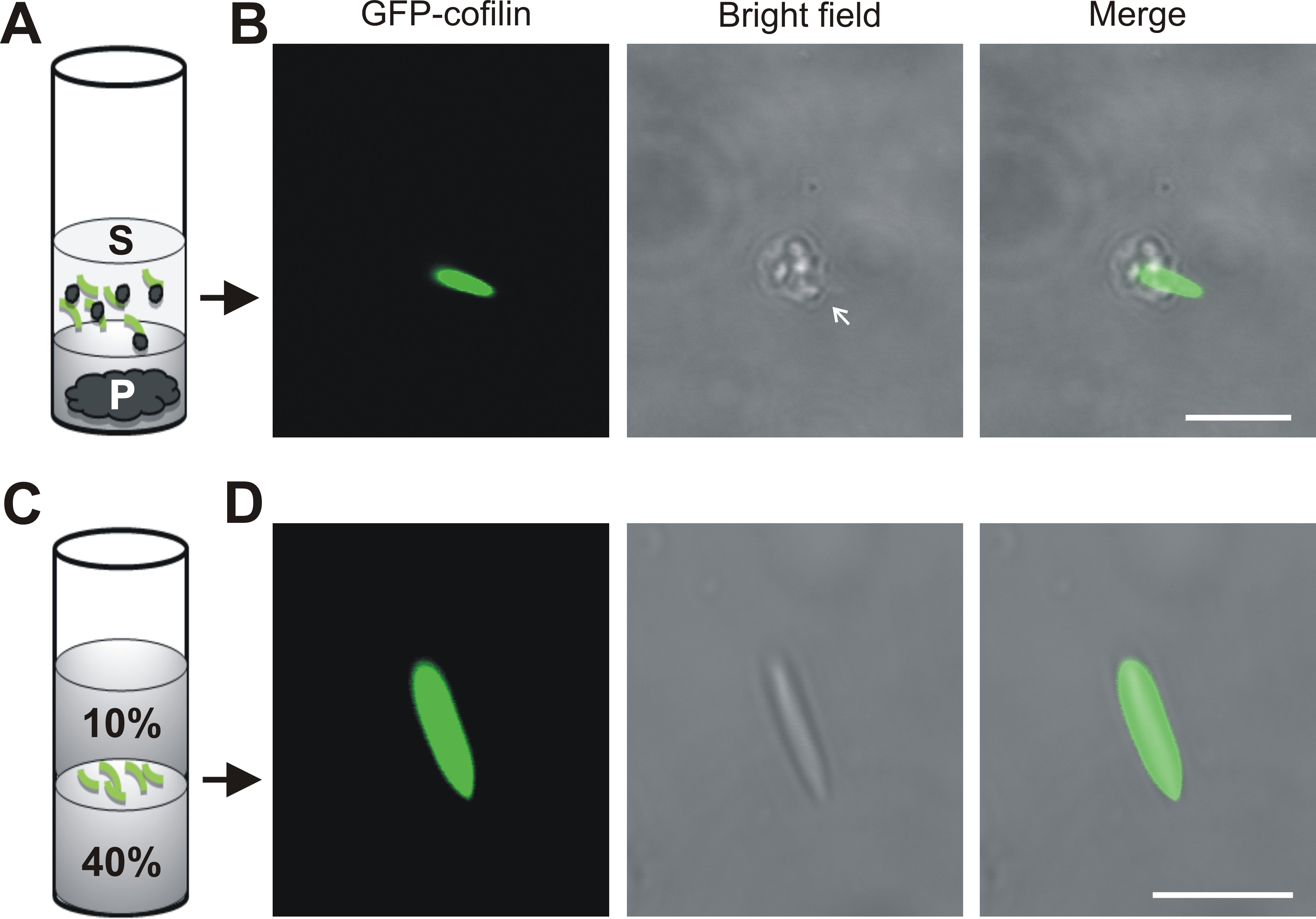
**

**Supplementary Figure 2. Purification of nuclear actin rods.** GFP-cofilin expressing *Dictyostelium* cells were induced to form nuclear rods by DMSO-treatment as described in Methods. The cell pellet was suspended in NP-40 lysis buffer, and the suspension was passed twice through nucleopore filters (5 µm pore size) to separate nuclei. (**a**) The filtrate was centrifuged, and the pellet fraction (P) contained cellular debris and some aggregated rods, the supernatant (S) the enriched rods. (**b**) The microscopic analysis showed that rods enriched in the supernatant were associated with nuclear debris as indicated by the arrow. (**c**) To optimize the purification, rods enriched in the supernatant were further purified by a two-step Optiprep gradient fractionation of 10 and 40% (w/v). Rods were collected from the interface between the 10 and 40% Optiprep layers. (**d**) Purity of isolated rods was checked by phase contrast and fluorescence microscopy. Scale bars are 5 μm.


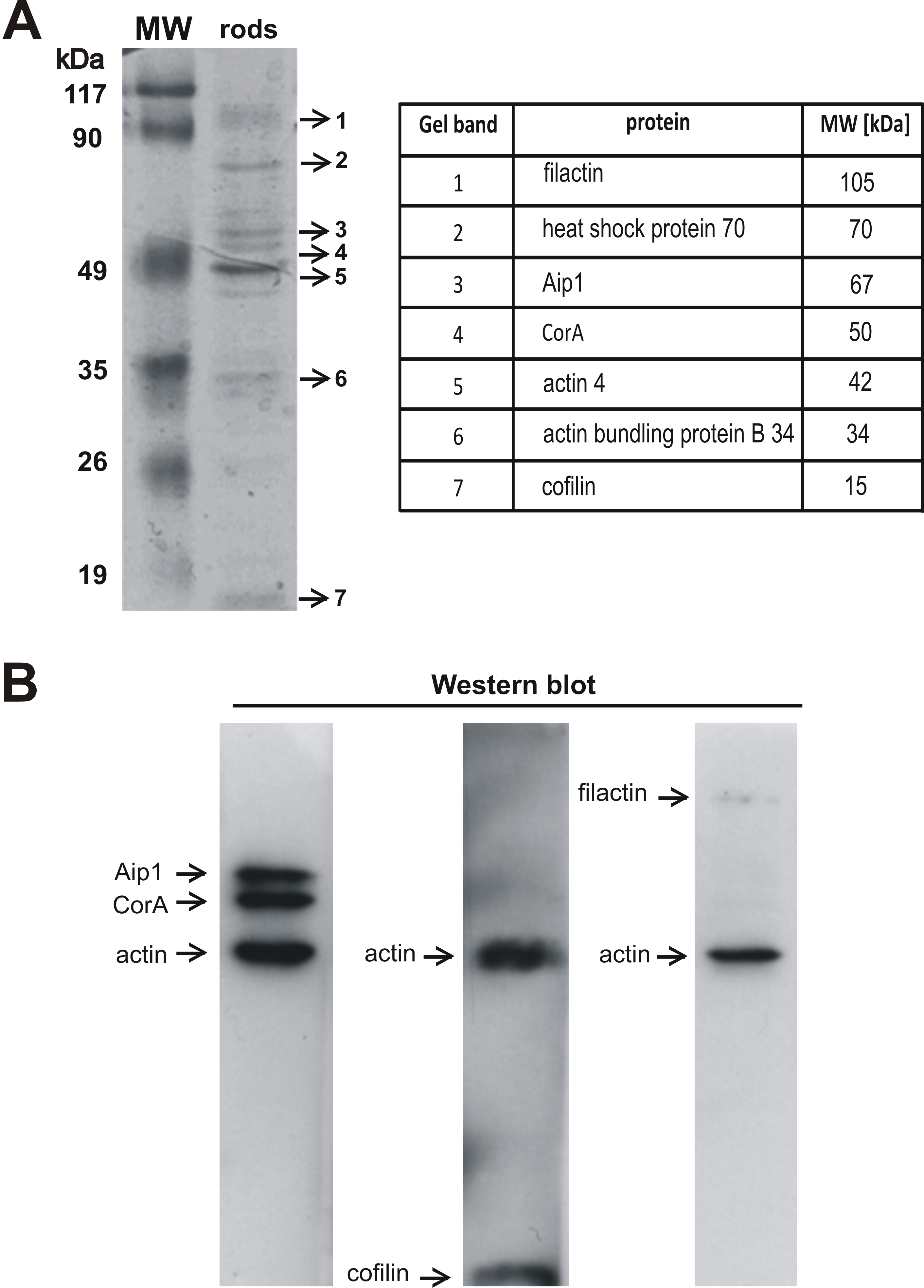


**Supplementary Figure 3.** **Identification of rod constituents by mass spectrometry and Western blot analysis**. (**a**) Samples of isolated rods were analysed by SDS-PAGE and Coomassie brilliant blue-R staining. Visible bands indicated by numbers (1-7) were cut out from the gel and analysed by mass spectrometry. The most abundant proteins identified from the individual bands by mass spectrometry are summarized in the table. Positions of molecular weight markers (MW) are indicated. (**b**) Western blot analysis of purified nuclear rod samples using antibodies against actin, Aip1, CorA, cofilin, and filactin.

**
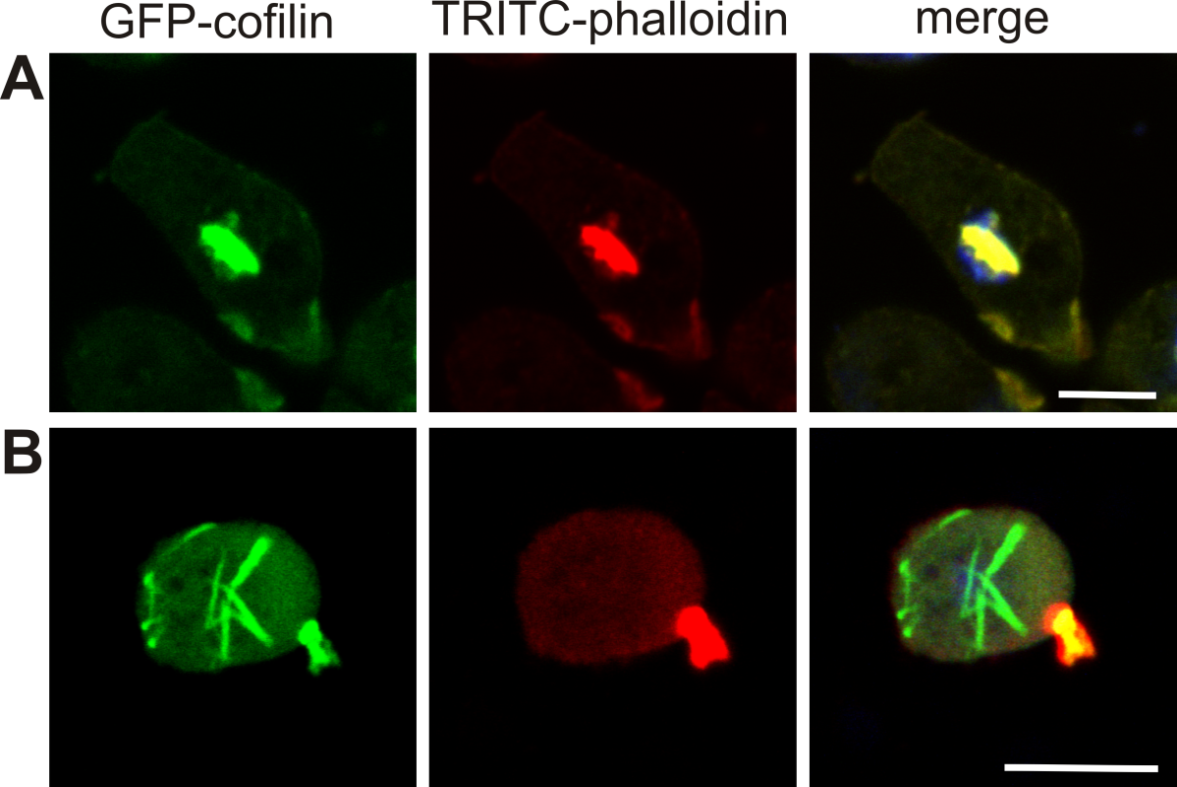
**

**Supplementary Figure 4. Only intranuclear but not cytoplasmic actin rods are stained by phalloidin.** (**a**) *Dictyostelium* cells expressing GFP-cofilin (green) were induced to form nuclear actin rods by treatment with 5% DMSO, and were fixed after 60 minutes. (**b**) Cytoplasmic actin rods were induced by addition of 10 mM (final concentration) sodium azide to the culture medium. Staining with TRITC-labelled phalloidin (red) visualizes only intranuclear rods and cortical actin structures, but not cytoplasmic rods. Scale bars are 10 µm.


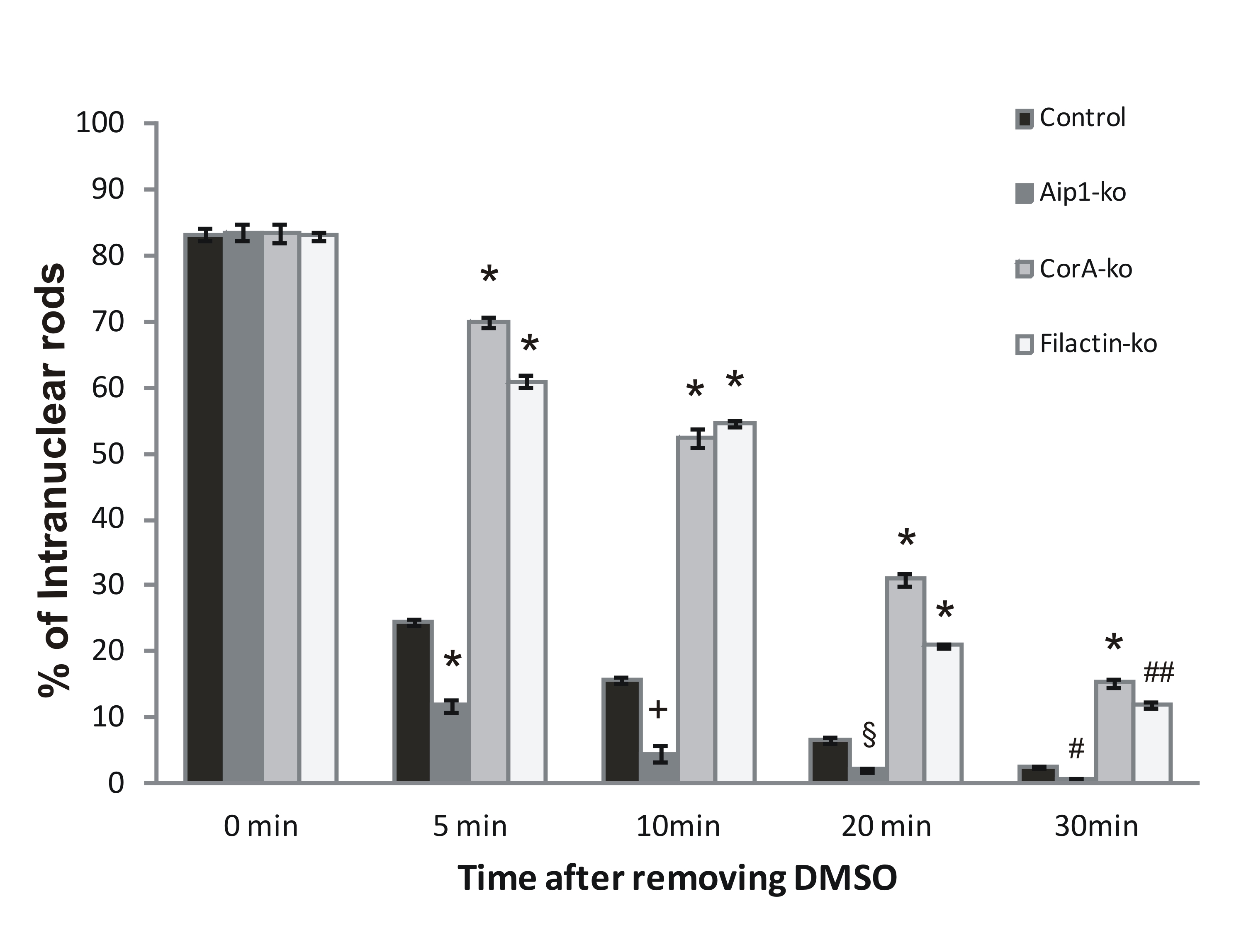


**Supplementary Figure 5. Time course of intranuclear rod disassembly.** *Dictyostelium* AX2 wild- type (control), or Aip1-ko, CorA-ko and Fia-ko cells were induced for 1 h to form intranuclear rods. Then (0 min), cells were washed three times with medium to remove the DMSO, and fixed after 5, 10, 20 and 30 minutes. After removal of the DMSO, four coverslips per strain were fixed for each time point, and the samples were examined and counted in at least 10 counting areas (>50 cells per counting area) per coverslip. The experiment was conducted three times. Percentage of cells with intranuclear rods was plotted. In cells lacking either CorA or Fia, rod disassembly is significantly delayed at all time points in comparison with the control. * p < 0.0001 and ## p= 0.0005. In Aip1-ko cells, rod disassembly is significantly faster than in control cells at all time points. + p=0.0003, § p=0.0006, # p= 0.0004. Data are presented as mean ±S.E.M. Statistical significance by unpaired t-test two-tailed is shown, and p< 0.05 was considered as significant. Errors bars indicate standard errors. GraphPad PRISM software was used for the statistical analysis.

**
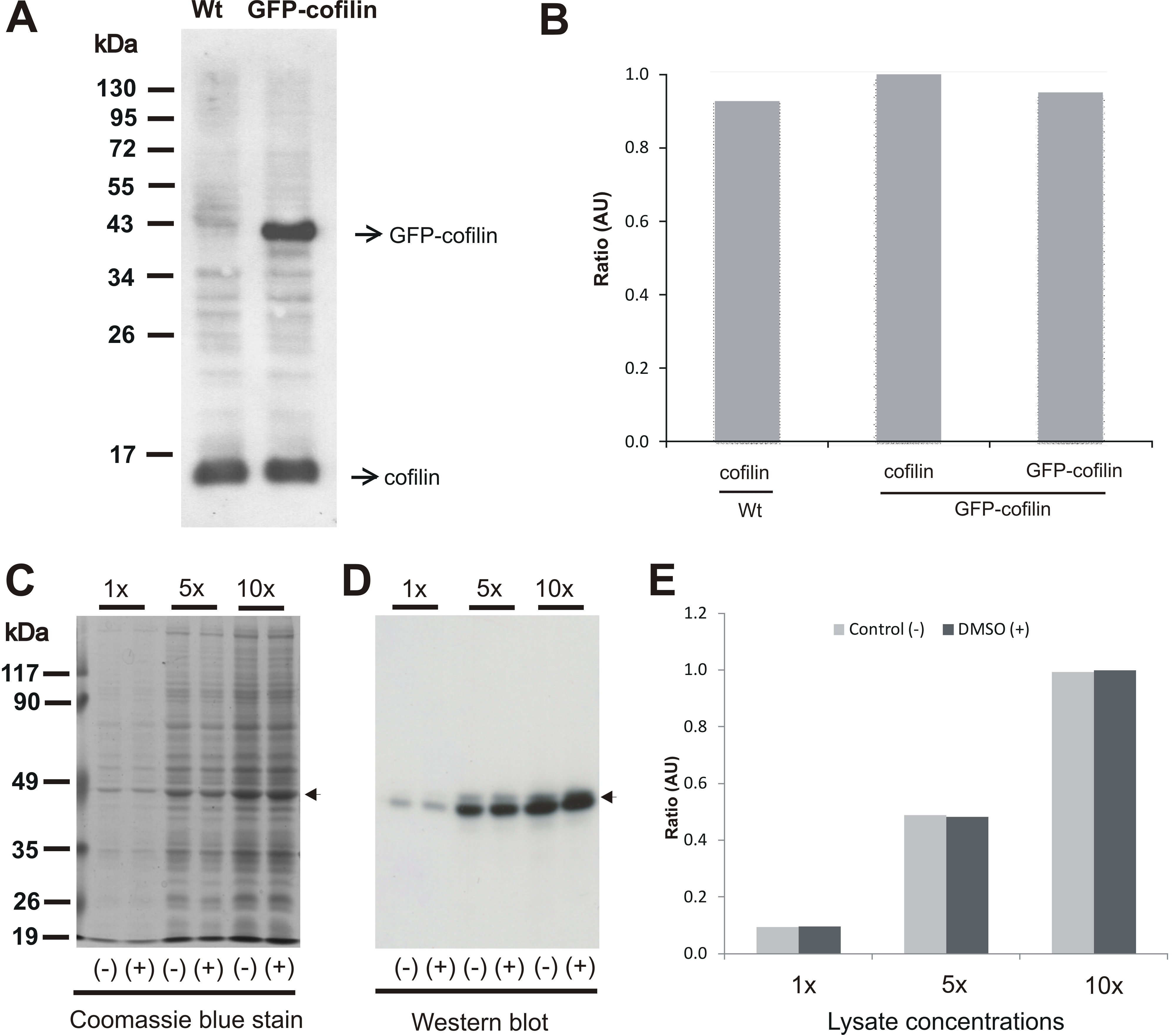
**

**Supplementary Figure 6. Endogenous cofilin and heterologously expressed GFP-cofilin have approximately equal protein expression levels, and expression levels are not changed by DMSO treatment.** (**a**) Samples of wild-type (wt) and GFP-cofilin expressing cells were analysed by Western blotting using anti-cofilin antibodies. Equal cell equivalents (2.5x10^5^) were applied per lane. (**b**) Quantitative analysis of protein expression. The levels of endogenous cofilin (wt), cofilin and GFP-cofilin are normalized to actin and represented in the histogram. (**c**) Lysates of GFP-cofilin expressing cells corresponding to 5x10^4^ (1x), 2.5x10^5^ (5x), and 5x10^5^ cells (10x), without (-) and with (+) DMSO-treatment to induce rod formation, were separated in a 10% gel and stained with Coomassie brilliant blue-R. The arrow indicates the position of actin which was used for normalization. (**d**) Western blot analysis of the samples shown in (c) using anti-GFP antibodies. The arrow indicates the position of GFP-cofilin. (**e**) Densitometric analysis of GFP-cofilin expression in control (-) and DMSO-treated (+) cells after normalization using actin as reference. Expression levels of GFP-cofilin do not significantly change after treatment with DMSO. Image J software was used to quantify protein levels from the Coomassie blue-stained gel and the Western blot. Data are represented as mean ±S.E.M.

**Legends to Supplementary Movies**

**Movie 1. Intranuclear actin rod formation of a cell expressing GFP-cofilin.** Live-cell imaging of a *Dictyostelium* cells expressing GFP-cofilin. Intranuclear rod formation was induced by treatment with 5% DMSO. The time series was recorded by confocal microscopy.

**Movie 2. Animated movie of a 3D reconstruction of the middle stage of actin rod formation.** The cell expressed GFP-cofilin (green), and was fixed after 20 min and subsequently stained with TO-PRO3 to visualize DNA (blue). The forming actin rod is surrounded by chromosomal DNA.

**Movie 3. Movie of a 3D reconstruction of the late stage of actin rod formation.** The cell expressed GFP-cofilin (green), and was fixed after 60 min and subsequently stained with TO-PRO3 to visualize DNA (blue). The bar-shaped actin rod is surrounded by chromosomal DNA.

**Movie 4. Animated movie showing actin nucleation along an isolated intranuclear rod.** Intranuclear rods were isolated from cells expressing GFP-cofilin (green) after 60 min of treatment with 5% DMSO. An actin polymerization assay was performed as described in Methods. Images were taken by confocal microscopy. The 3D reconstruction shows that actin (red) polymerizes along the rod (green) core sides and ends.

**Movie 5. Animated movie showing actin nucleation from the core side of an isolated intranuclear rod.** Intranuclear rods were isolated from cells expressing GFP-cofilin (green) after 60 min of treatment with 5% DMSO. An actin polymerization assay was performed as described in Methods. Images were taken by confocal microscopy. The 3D reconstruction shows two actin bundles originating from the actin-cofilin rod core side and indicates that actin (red) can polymerize from the lateral sides of the rod (green).
